# Adolescents’ Mindfulness and Psychological Distress: The Mediating Role of Emotion Regulation

**DOI:** 10.3389/fpsyg.2019.01358

**Published:** 2019-06-07

**Authors:** Ying Ma, Siqi Fang

**Affiliations:** ^1^School of Education, Shaanxi Normal University, Xi’an, China; ^2^Faculty of Education, The Chinese University of Hong Kong, Sha Tin, Hong Kong

**Keywords:** mindfulness, difficulties in emotion regulation, cognitive reappraisal, expressive suppression, psychological distress, adolescents

## Abstract

Mindfulness has been widely linked with psychological well-being in general population. There are emerging studies supporting the relationship between adolescents’ mindfulness and their mental health. However, the mechanisms through which mindfulness may influence adolescents’ psychological distress have only recently been explored, and more related research is still needed. This study investigated the relationship between adolescents’ dispositional mindfulness and psychological symptoms of depression, anxiety and stress. The mediating variables were also explored in perspective of two common emotion regulation theories, which were measured through Difficulties in Emotion Regulation Scale (DERS) and Emotion Regulation Questionnaire (ERQ). DERS has been used as a comprehensive assessment of emotion regulation difficulties. ERQ is also widely accepted to measure the emotion regulation process including dimensions of cognitive reappraisal and expressive suppression. Measures assessing mindfulness, emotion regulation, and psychological distress were administered to 1067 adolescents in mainland China. The results confirmed that adolescents’ dispositional mindfulness was negatively associated with depression, anxiety, and stress. DERS, especially the sub-dimensions of Acceptance and Strategies, significantly mediated the relationship between mindfulness and symptoms of depression, anxiety and stress. Whereas, ERQ including subscales of cognitive reappraisal and expressive suppression exerted limited mediating effect. These findings provided insights for the potential underlying mechanism between adolescents’ mindfulness and psychological distress, demonstrating that DERS might be more pervasive than ERQ. Further research was suggested to explore other mediating variables underlying mindfulness and psychological distress among adolescents and develop mindfulness-based programs to improve adolescents’ mindfulness and emotion regulation ability.

## Introduction

Adolescence is a turbulent period fraught with numerous developmental changes and may be vulnerable for psychological distress (Silk et al., 2003; Zeman et al., 2006). Since various psychopathologies, including depression, anxiety, and behavioral disorders can dramatically increase during adolescence (e.g., McLaughlin et al., 2011). Anxiety and depression as the most commonly mental disorders might induce many aspects of health problems such as affective, cognitive, neurovegetative symptoms and hypertension (Mucci et al., 2016a). Therefore, it is critical to understand the potential protective factors which might reduce adolescents’ psychological distress. Dispositional mindfulness is considered as one of the most prominent factors due to its relations with various mental health outcomes (Brown and Ryan, 2003; Brown et al., 2007).

Mindfulness was defined as an experience that can bring attention to the present moment with non-judgmental attitude (Kabat-Zinn, 1990). Evidence has demonstrated that dispositional mindfulness is significantly associated with psychological distress among adults (e.g., Jimenez et al., 2010; Soysa and Wilcomb, 2015). It is also suggested that levels of dispositional mindfulness vary among individuals. Specifically, high levels of mindfulness are positively associated with a variety of aspects concerning well-being, such as positive emotions (Jimenez et al., 2010), life satisfaction (Shonin et al., 2013), and overall health (Allen and Kiburz, 2012); whereas low levels of mindfulness are associated with depression, anxiety, and stress (Jimenez et al., 2010; Soysa and Wilcomb, 2015). In addition, mindfulness can be improved through special trainings. Abundant research has demonstrated that mindfulness-based intervention can effectively reduce psychological distress and promote mental health outcomes (Kabat-Zinn, 1990; Segal et al., 2002; Brown and Ryan, 2003).

Nevertheless, there is a dearth of empirical research on adolescents’ mindfulness. Most efforts of the limited studies have focused on examining the effectiveness of mindfulness-based intervention among clinical adolescent samples (Bluth and Blanton, 2014). Previous studies have confirmed that mindfulness practice could help adolescents reduce chronic pain, alcohol addiction and inflammatory bowel disease (Harris et al., 2017; Ruskin et al., 2017; Kohut et al., 2019). Mindfulness mediation could be an effective and immediate intervention to help reduce adolescents’ state anxiety on an inpatient psychiatric unit (Blum et al., 2019). While, more evidence is still needed to investigate the generalizability of these results from intervention studies, particularly among non-clinical adolescent samples (Tan, 2016). Furthermore, the results of mindfulness-based intervention studies can’t be directly generalized to the research on adolescents’ dispositional mindfulness (Tan, 2016). The present study aims to fill this gap by examining the proposed negative relationship between adolescents’ dispositional mindfulness and psychological distress in a normal adolescent sample, and further exploring the potential underlying mechanisms.

Researchers originally proposed a mindfulness model that mainly focused on the two components of (1) self-regulation of attention, which refers to non-judgmental awareness of sensations, thoughts, and feelings in the present moment in “a beginner’s mind,” and (2) adoption of a particular orientation that is characterized by curiosity, openness, and acceptance toward one’s experience (Bishop et al., 2004). Mindfulness emphasizes the awareness of mental and emotional process, as well as promotes the attitudes of curiosity, patience, and non-judgment toward distress, which may reduce adolescents’ tendencies to ruminate on negative events or emotions and serve as protective factor against psychological distress (Shapiro et al., 2006). Individuals with high level of mindfulness are more likely to observe, understand and accept the negative emotions with upsetting experiences, instead of being occupied with avoiding or rejecting these emotions (Cheung and Ng, 2019). Thus, in considering the benefits of mindfulness in reducing adolescents’ psychological distress, emotion regulation is likely to be an important construct that linked mindfulness with psychological distress (Pepping et al., 2013).

Emotion regulation refers to the ability to effectively manage the affective states whereas the lack of which is identified as a critical factor in many psychological problems (Gross, 1998; Zeman et al., 2006). Specifically, emotion regulation ability is crucial for adolescents’ emotional experiences and their adaptation to socioemotional challenges (Silk et al., 2003; McLaughlin et al., 2011). Although recent research has attempted to explain the association between mindfulness and psychological distress (Pepping et al., 2013; Freudenthaler et al., 2017), the potential role of emotion regulation in between and its underlying mechanisms is still not clear, especially among adolescents. To this end, the present study will explore the mediating roles of two common emotion regulation models, which are Difficulties in Emotion Regulation (Gratz and Roemer, 2004) and Emotion Regulation Process (Gross and John, 2003) models.

Gratz and Roemer (2004) proposed an integrative conceptualization of emotion regulation which assumes that emotion regulation involves not only the modulation of emotional arousal, but also the awareness, clarity, and acceptance of emotions, as well as the ability to control emotional impulse and act in a desired way. Adaptive emotion regulation includes a range of emotion regulation strategies and the flexibility of using them. The lack of these abilities may result in emotion dysregulation. Based on Gratz and Roemer’s (2004) model of emotion regulation, the Difficulties in Emotions Regulation Scale (DERS) was developed accordingly for the measurement of the difficulties in emotion regulation. DERS has been confirmed valid in assessing adolescents’ emotion regulation and demonstrated to be related to adolescents’ externalizing and internalizing problems (Neumann et al., 2010). Recent studies have provided initial evidence about the mediating role of DERS between mindfulness and psychopathology. For example, the study of Ma et al. (2018) found that an online mindfulness-based intervention could help reduce psychological distress in a normal population, and DERS could mediate the relationship between changes in mindfulness and changes in psychological distress. However, DERS was typically tested as a unidimensional phenomenon and few researches have examined its dimensions simultaneously as different mediators between mindfulness and psychological well-being. Therefore, we aim to fill this gap in exploring different mediating effects of DERS multidimensionally among adolescents.

In the meantime, the Emotion Regulation Process (Gross, 1998) is another common theory in the field of emotion regulation which emphasizes antecedent-focused and response-focused strategies of emotion regulation process. Gross (1998) defined emotion regulation as “the processes by which individuals influence which emotions they have, when they have them, as well as how they experience and express these emotions.” This information-processing model treats each step in the emotion-generating process as a potential target strategy of emotion regulation process. This process includes five main points: situation selection, situation modification, deployment of attention, cognitive change, and response modulation. The five points of emotion regulation process are classified as antecedent-focused strategies and response-focused strategies. Antecedent-focused strategies refer to those that modify the input of emotion prior to the actual emotional experience, while response-focused strategies refer to those that alter emotional responses after the emotions have been fully elicited (Gross and John, 2003). Later on, the Emotion Regulation Questionnaire (ERQ) was developed to assess the emotion regulation process including two dimensions of cognitive reappraisal and expressive suppression (Gross and John, 2003). Cognitive reappraisal is the most typical antecedent-focused emotion regulation strategy. It emphasizes changing one’s thoughts about a potential emotion-eliciting situation to avoid a negative emotional impact or facilitating a positive emotional experience (Gross and John, 2003). It usually occurs at the earlier stage of the emotion regulation process and plays a role prior to full arousal of emotional response tendencies (Gross, 1998; Gross and John, 2003). Cognitive reappraisal has been demonstrated to be an adaptive emotion regulation skill and a protective factor for emotional well-being (Gross and John, 2003; Gross and Jazaieri, 2014). In contrast, expressive suppression as a typical response-focused strategy occurs relatively late in the emotion-generating process. It is found that suppressors are less clear about their feelings and less successful in recovering their mood (Gross and John, 2003). As such, expressive suppression is usually regarded as a maladaptive strategy that can lead to overwhelming emotions, and in turn results in experiences of psychological distress such as depression (Gross and John, 2003; Gross and Jazaieri, 2014).

In terms of the mediating role of emotion regulation between mindfulness and psychological distress, Gross (2014) proposed that mindfulness may promote the cognitive reappraisals and facilitates the attitudes of acceptance in opposite to suppression. Individuals with high levels of mindfulness are more likely to apply cognitive reappraisal strategies because they may have more ability of metacognition, including monitoring of the consciousness and control of the cognitive processes (Bishop et al., 2004). In other words, with higher level of mindfulness, individuals are easier to pause before they directly react to negative emotions. Additionally, they are more likely to disengage from automatic thoughts and focus on the present moment experience (Bishop et al., 2004). This allows individuals to have more insights into their own emotional appraisal and to notice their automatic reactions that are always triggered by certain situations, so that they may effectively regulate their own emotions and select a rational reappraisal to deal with the situations (Gross and Jazaieri, 2014). Meanwhile, mindfulness also emphasizes an attitude of acceptance without positive or negative judgments (Shapiro et al., 2006; Brown et al., 2007). Individuals with high levels of mindfulness are more likely to notice and accept all experiences without denying or suppressing frustrated feelings. Studies have shown that higher level of acceptance is associated with lower level of suppression (Liverant et al., 2008). Research findings indicated that expressive suppression mediated the association between mindfulness and psychological distress, whereas cognitive reappraisal did not show significant result (Pepping et al. (2016). Due to the limited sample of this study, more evidence is required to support the mediating roles of cognitive reappraisal and expressive suppression between adolescents’ mindfulness and psychological distress in a lager sample from different cultures.

In summary, Difficulties in Emotion Regulation and Emotion Regulation Process as two emotion regulation models target at explaining different aspects underlying emotion regulation. Difficulties in Emotion Regulation focuses more on individual’s inherent general capacity of emotion regulation, whilst Emotion Regulation Process tends to emphasize the dynamic process of emotion regulation. The present study was designed to explore the mediating effects of two emotion regulation models simultaneously, which could provide a more comprehensive understanding of the associations between emotion regulation, mindfulness, and psychological distress in adolescents. We hypothesize that adolescents who have a higher level of dispositional mindfulness would experience less psychological distress, and this relationship would be mediated by DERS, and cognitive reappraisal and expressive suppression of ERQ.

## Materials and Methods

### Participants and Procedure

The questionnaires were distributed to middle school students after receiving ethics approval from the Chinese University of Hong Kong. We got the permission through the school principal and teachers before the survey. Both parents’ and adolescents’ consents were collected before the study. Then the questionnaires were completed in the classroom during school hours and returned to the researchers via teachers of the school. All participants completed their questionnaires on a voluntary basis.

A total of 1200 questionnaires were distributed, and 1067 participants completed the questionnaire. The response rate was about 89% which is satisfactory. The participants were ranging from 12 to 18 years old from four middle schools in Chinese mainland. The average age was 14.84 (*SD* = 1.59) with 57.8% female. There were 229 participants (21.4%) from grade 7, 263 participants (24.4%) from grade 8, 98 participants (9.2%) from grade 9, 195 participants (18.2%) from grade 10, 113 participants (10.6%) from grade 11, 173 participants (16.2%) from grade 12.

### Measures

The Mindful Attention Awareness Scale (MAAS; Brown and Ryan, 2003) was used to measure adolescents’ dispositional mindfulness. MAAS is a single factor scale with 15 items using a 6-point Likert-type scale (from “almost always” to “almost never”). It has been translated into Chinese and verified in Chinese adolescents (Black et al., 2012). The internal consistency of this scale in the present study was 0.78.

Depression Anxiety Stress Scales (DASS; Lovibond and Lovibond, 1995) is a 21-item measure consisting of three subscales: depression, anxiety, and stress. Items were scored on a four-point scale ranging from 0 (“did not apply to me at all”) to 3 (“applied to me very much or most of the time”). Each subscale’s total score is calculated as the sum of its corresponding item scores (multiplied by two). Higher scores indicate greater depression, anxiety, or stress. It reflects a common “negative affect” as well as three distinct syndromes of anxiety, depression and stress. Previous studies have applied DASS to assess the general psychological distress in the sample of adolescents (Wang et al., 2011). The factor structure of the Chinese version of DASS-21 was also supported by previous studies and the internal consistency of the subscales was above 0.70 (Wang et al., 2011). In the present study, Cronbach’s alphas of subscales of DASS-21 were 0.80 (depression), 0.76 (anxiety), 0.72 (stress).

Difficulties in Emotion Regulation Scale (DERS) (Gratz and Roemer, 2004) consists of 36 items scored on a five-point scale ranging from 1 (almost never) to 5 (almost always). The original version included six dimensions in total. However, previous study found that the dimension of Awareness shared only the modest correlation with other subscales of DERS, and it didn’t show the same higher-order construct with other five dimensions (Bardeen et al., 2012). Therefore, the dimension of Awareness was suggested to be excluded in the revised DERS (Bardeen et al., 2012). Thus, the five dimensions of DERS, including lack of emotional clarity (Clarity), difficulty in engaging in goal-directed behavior under negative emotions (Goals), loss of control under negative emotions (Impulse), limited strategies for emotion regulation (Strategies), and non-acceptance of emotional responses (Non-acceptance), were adopted in the present study. The Cronbach’s alphas of the subscales ranged from 0.71 to 0.84 in this study.

Emotion Regulation Questionnaire (Gross and John, 2003) to assess the emotion regulation process. It consists of two subscales of cognitive reappraisal and expressive suppression. Items are rated on a five-point scale from 1 (strongly disagree) to 5 (strongly agree). ERQ has been used in many previous studies with good validity and reliability (e.g., Pepping et al., 2016). In the present study, the Cronbach’s alphas of cognitive reappraisal and expressive suppression subscales were 0.70 and 0.63, respectively.

### Concerns for Common Method Bias

The statistical methods were used to address the concern about common method bias. All the variables were loaded into an exploratory factor analysis (EFA). If a substantial amount of common method bias exists, a single factor will emerge from the factor analysis or account for the majority of the covariance (Podsakoff, 2003). Results of EFA showed that 17 components with eigenvalues > 1.0 were identified. These 17 factors accounted for 55.13% of the variance. The largest component accounted for 18.91% of the variance. These results suggest that common method bias is not likely a major concern in the current study (Podsakoff, 2003).

### Data Analysis

Description data such as Means and SDs of variables were analyzed and then Pearson’s correlations among variables were conducted through SPSS 20.0. The mediating roles were explored using a bootstrapping procedure of 5,000 samples via SPSS process (Preacher and Hayes, 2008; Hayes and Preacher, 2013). This process would generate a 95% confidence interval to test the significance of the indirect effect of the mindfulness on the depression, anxiety, and stress through the proposed mediators including the DERS total score and subscale scores, cognitive reappraisal and expressive suppression. The indirect effects are considered significant when zero is not included within the lower and upper confidence intervals (CIs).

## Results

Means, SDs, and Pearson’s correlations for studied variables are displayed in Table 1. Adolescents’ dispositional mindfulness was negatively related to depression, anxiety, and stress. Mindfulness was also negatively related to the total score of DERS and the scores of its subscales. In terms of ERQ, mindfulness was negatively related to expressive suppression, whereas non-significant relationship was found between mindfulness and cognitive reappraisal.

**Table 1:** Descriptive statistics and correlations between all studied variables.

	Mean (SD)	1	2	3	4	5	6	7	8	9	10	11	12
1. Mindfulness	3.93 (0.76)	1											
2. Reappraisal	3.39 (0.65)	0.05	1										
3. Suppression	2.86 (0.80)	-0.18^**^	0.10^**^	1									
4. DERS	2.08 (0.52)	-0.52^**^	-0.20^**^	0.05	1								
5. Clarity	2.45 (0.75)	-0.30^**^	-0.18^**^	0.08^*^	0.54^**^	1							
6. Goals	3.12 (0.90)	-0.47^**^	-0.09^**^	0.01	0.75^**^	0.22^**^	1						
7. Impulse	2.39 (0.89)	-0.39^**^	-0.13^**^	-0.02	0.85^**^	0.31^**^	0.51^**^	1					
8. Strategies	3.53 (0.80)	-0.44^**^	-0.19^**^	0.09^**^	0.88^**^	0.32^**^	0.55^**^	0.68^**^	1				
9. Acceptance	3.53 (0.85)	-0.36^**^	0.03	0.15^**^	0.58^**^	0.27^**^	0.38^**^	0.48^**^	0.58^**^	1			
10. Depression	12.11 (8.29)	-0.50^**^	-0.15^**^	0.22^**^	0.60^**^	0.31^**^	0.43^**^	0.47^**^	0.60^**^	0.45^**^	1		
11. Anxiety	14.31 (8.22)	-0.50^**^	-0.05	0.14^**^	0.57^**^	0.24^**^	0.39^**^	0.51^**^	0.56^**^	0.51^**^	0.67^**^	1	
12. Stress	17.46 (8.00)	-0.46^**^	-0.06	0.09^**^	0.62^**^	0.24^**^	0.47^**^	0.52^**^	0.60^**^	0.50^**^	0.65^**^	0.73^**^	1


*N = 1063. ^∗^*p* < 0.05; ^∗∗^*p* < 0.01. DERS = Difficulties in Emotion Regulation Scale (DERS).*

**FIGURE 1:**
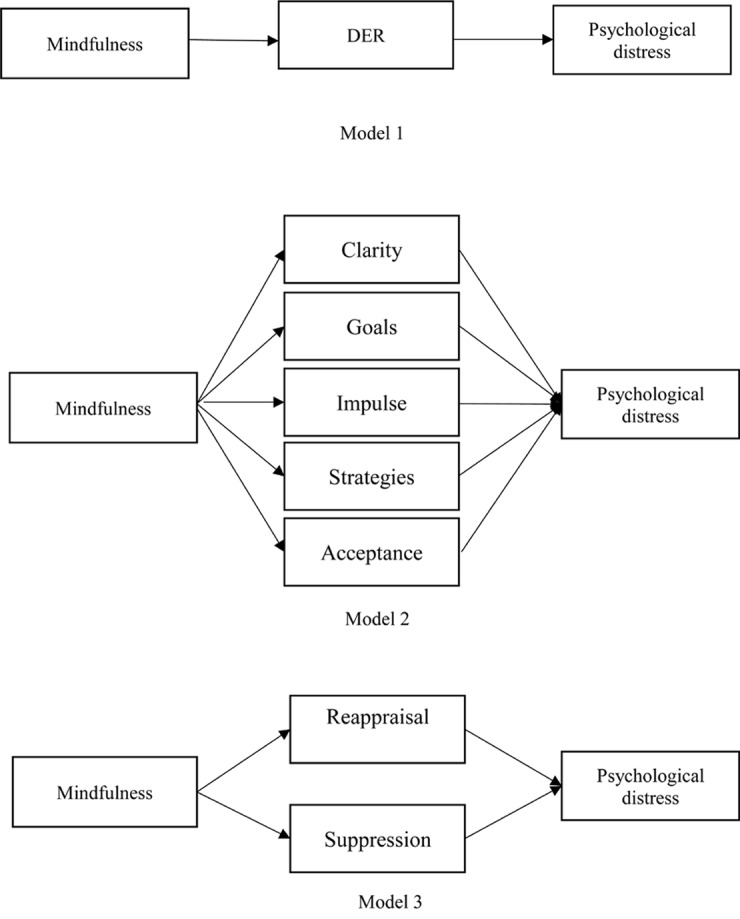
Conceptual mediation model of the presumed influence of mindfulness on psychological distress. Model 1 with the DERS total score as mediating variable. Model 2 with the DERS subscales as the mediating variables. Model 3 with emotion regulation process (cognitive reappraisal and expressive suppression) as the mediating variable.

Figure 1 showed three different conceptual mediation model of the presumed influence of mindfulness on psychological distress. Model 1 with the DERS total score as mediating variable. Model 2 with the DERS subscales as the mediating variables. Model 3 with emotion regulation process (cognitive reappraisal and expressive suppression) as the mediating variable. In model 1, we set DERS total score as mediating variables. The direct effect of mindfulness on depression (β = –0.21, 95% CI = [–0.25, –0.16], *p* < 0.001), anxiety (β = –0.21, 95% CI = [–0.26, –0.17], *p* < 0.001) and stress (β = –0.14, 95% CI = [–0.18, –0.10], *p* < 0.001) were significant. The indirect effect of mindfulness on depression (β = –0.24, 95% CI = [–0.28, –0.20], *p* < 0.001), anxiety (β = –0.22, 95% CI = [–0.26, –0.19], *p* < 0.001) and stress (β = –0.27, 95% CI = [–0.31, –0.24], *p* < 0.001) were significant (Table 2). Model 1 predicted 48% of the variance in depression, and 45 and 54% of the variance in anxiety and stress, respectively.

**Table 2:** Indirect effects of mindfulness on depression, anxiety and stress through different mediating variables.

	Depression			Anxiety			Stress		
			
	β	95% CI	*p*	β	95% CI	*p*	β	95% CI	*p*
**Model 1**	-0.24	–0.28, –0.20	<0.001	-0.22	–0.26, –0.19	<0.001	-0.27	–0.31, –0.024	<0.001
**Model 2**	-0.25	–0.29, –0.21	<0.001	-0.22	–0.26, –0.19	<0.001	-0.28	–0.32, –0.24	<0.001
Clarity	-0.02	–0.04, –0.01	<0.01	0.01	–0.01, 0.02	0.39	0.004	–0.01, 0.02	0.56
Goals	-0.02	–0.05, 0.01	0.22	0.01	–0.02, 0.04	0.45	-0.05	–0.08, –0.02	<0.001
Impulse	-0.01	–0.04, 0.02	0.43	-0.07	–0.10, –0.04	<0.001	-0.05	–0.08, –0.02	<0.001
Strategies	-0.16	–0.20, –0.12	<0.001	-0.09	–0.12, –0.06	<0.001	-0.12	–0.16, –0.09	<0.001
Acceptance	-0.04	–0.06, –0.02	<0.001	-0.08	–0.11, –0.06	<0.001	-0.06	–0.09, –0.04	<0.001
**Model 3**	-0.04	–0.05, –0.02	<0.001	-0.01	–0.02, –0.001	0.06	-0.004	–0.02, 0.01	0.52
Reappraisal	-0.01	–0.02, 0.002	0.14	-0.001	–0.01, 0.001	0.41	-0.002	–0.01, 0.001	0.41
Suppression	-0.03	–0.04, –0.02	<0.001	-0.01	–0.02, 0.00	0.07	-0.002	–0.01, 0.01	0.67


*Model 1 with the DERS total score as mediating variable. Model 2 with the DERS subscales as the mediating variables. Model 3 with emotion regulation process (cognitive reappraisal and expressive suppression) as the mediating variable. The values reported on the same row of “Model 1”, “Model 2” and “Model 3” refer to the total indirect effects.*

In Model 2, the direct effect of mindfulness on depression (β = –0.20, 95% CI = [–0.24, –0.16], *p* < 0.001), anxiety (β = –0.21, 95% CI = [–0.25, –0.17], *p* < 0.001) and stress (β = –0.14, 95% CI = [–0.18, –0.10], *p* < 0.001) were significant. Regarding DERS subscales as mediating variables, the total indirect effect of mindfulness on depression (β = –0.25, 95% CI = [–0.29, –0.21], *p* < 0.001), anxiety (β = –0.22, 95% CI = [–0.26, –0.19], *p* < 0.001) and stress (β = –0.28 95% CI = [–0.32, –0.24], *p* < 0.001) were significant (Table 2). Model 2 predicted 49% of the variance in depression, and 45 and 60% of the variance in anxiety and stress, respectively. Dimensions of Clarity, Strategies and Acceptance exerted significant mediating effects on depression. Dimensions of Impulse, Strategies and Acceptance exerted significant mediating effects on anxiety. Moreover, dimensions of Goals, Impulse, Strategies and Acceptance exerted significant mediating effects on stress (Table 2).

In Model 3, with ERP (cognitive reappraisal and expressive suppression) as mediating variables, the total indirect effect of mindfulness on depression (β = –0.03, 95% CI = [–0.05, –0.02], *p* < 0.001) was significant. However, the total indirect effect of mindfulness on anxiety (β = –0.01, 95% CI = [–0.01, 0.00], *p* = 0.06) and stress (β = –0.004, 95% CI = [–0.02, 0.01], *p* = 0.52) were not significant. The direct effect of mindfulness on depression (β = –0.36, 95% CI = [–0.41, –0.33], *p* < 0.001), anxiety (β = –0.38, 95% CI = [–0.42, –0.33], *p* < 0.001) and stress (β = –0.34, 95% CI = [–0.39, –0.30], *p* < 0.001) were significant (Table 2). Model 3 predicted 3% of the variance in depression, and 2 and 1% of the variance in anxiety and stress, respectively. Dimension of expressive suppression exerted significant mediating effect on depression.

## Discussion

The aim of this study was to examine whether mindfulness would be negatively associated with adolescents’ depression, anxiety and stress, and whether DERS and ERQ would help to shed light upon the mechanisms underlying this relationship in between. Results revealed that adolescents’ mindfulness was negatively associated with psychological distress. This relationship was mediated by the total score and subscales of DERS. Regarding the ERQ, only expressive suppression showed a small mediating effect on the relationship between the mindfulness and the depression.

Our finding showed that mindfulness is negatively related to adolescents’ psychological distress, which echoes previous literature suggesting that mindfulness is positively related to the general health and life satisfaction, while negatively related to negative emotions such as depression and anxiety (Jimenez et al., 2010; Allen and Kiburz, 2012; Shonin et al., 2013). Results also indicated that the DERS total score mediated the relationship between mindfulness and psychological distress (depression, anxiety, and stress). Mindfulness tends to predict more emotional self-regulation ability because it is characterized with awareness and clarity of emotions, and acceptance without judgment were associated with less difficulties of emotion regulation (Jimenez et al., 2010; Vujanovic et al., 2010). In addition, high level of mindfulness may facilitate adaptive emotion regulation, reduce habitual reactivity to negative emotions, and enable effective and adaptive psychological responses (Arch and Craske, 2006), which in turn reduce distress outcomes. With low level of mindfulness, adolescents might be lack of emotion clarity, self-control and acceptance, which in turn might lead to their poor realization of emotion and easy immersion into dysfunctional emotional reactions such as impulse and aggressive behavior toward others or blame to themselves. All of these are associated with high level of psychological distress (Jimenez et al., 2010).

With regard to the mediating effects of the DERS subscales, results indicated that Strategies and Acceptance exerted more significant mediating effects comparing with others. This echoes findings on that the indirect associations of mindfulness with psychological distress and social difficulties were mediated by non-acceptance of emotions and lack of access to emotion regulation strategies (Pepping et al., 2014). Mindfulness is demonstrated to be significantly linked with the improvement of cognitive flexibility which may help get access to diverse strategies of emotion regulation (Moore and Malinowski, 2009). Therefore, the dimension of Strategies could serve as a mediator. Moreover, Carson and Langer (2006) proposed that mindfulness could improve self-acceptance by promoting the ability and willingness to maintain authenticity of self, since individuals with high levels of mindfulness pay more attention to the present feeling of the self and thus could avoid mindless pretending behavior. In other words, being mindful could help individuals to authentically accept themselves without thinking excessively about the evaluations of others. Another longitudinal study also confirmed the mediating role of difficulties in emotion regulation between mindfulness and symptoms of depression and anxiety in a sample of emerging adults. This study found that sub-dimensions of DERS such as acceptance of negative emotions could serve as mediators (Cheung and Ng, 2019). Mindfulness increases cognitive flexibility, which can help individuals to view past mistakes from both positive and negative perspectives, and thereby achieve unconditional self-acceptance (Carson and Langer, 2006). Thus, these can facilitate our understanding that the dimension of Acceptance significantly mediated the relationship between mindfulness and psychological distress.

Results also showed that mindfulness was negatively related to expressive suppression, while expressive suppression mediated the relationship between mindfulness and depression. However, cognitive appraisal was neither significantly related to mindfulness nor was its mediating effect significant between mindfulness and psychological. It is proposed that mindfulness may help facilitate a non-judgmental attitude of acceptance, which further improves individuals’ acceptance of their emotional experiences, and as a result, help to reduce expressive suppression (Gross, 2014). As such, mindfulness encourages an approach of openness, curiosity, and acceptance, which could help individuals to become less likely to avoid or suppress emotions, since both positive and negative emotions are perceived without judgment. Therefore, with higher level of mindfulness, adolescent might reduce expressive suppression of negative experience, which in turn help improve their mental well-being (Gross and Jazaieri, 2014). Regarding the relationship between mindfulness and cognitive reappraisal, Gross (2014) proposed that mindfulness may promote the cognitive reappraisals. Since it may help individuals keep some distance from their autonomic thoughts and focus the attention into the present moments, mindfulness tends to implicitly help individuals realize their negative autonomic thoughts and replace them with other positive thoughts. In contrast, Nyklíček (2011) has proposed that mindfulness emphasizes non-judgment of experiences, so that there is no need to reappraise the situation but to accept it. From this point of view, negative thoughts do not need to be replaced with positive thoughts. Mindfulness may not directly improve cognitive reappraisal but may instead improve de-appraisal and then induce positive cognition of the situation in a natural way. Troy et al. (2013) have also claimed that, although cognitive reappraisal is generally an adaptive emotion regulation strategy, the adaptive nature of reappraisal depends on context. When stressors are uncontrollable, the strategy of reappraisal is adaptive in regulating emotion by oneself. On the contrary, when individuals can control stressors, they may prefer other strategies, and reappraisal seems to not be adaptive. Additionally, Chambers et al. (2009) have argued that with higher levels of mindfulness, individuals may have more ability to access many other emotion regulation strategies aside from cognitive reappraisal. In short, mindfulness is more likely to facilitate a broad selection of strategies to regulate emotion. More specifically, rather than cognitive reappraisal, non-judgment and acceptance may be more theoretically consistent with the concept of mindfulness, which may help reduce expressive suppression.

All in all, the results indicated that DERS may be more appropriate for explaining the mechanism underlying mindfulness and psychological distress among adolescents. The possible reason may be that, comparing with the ERQ, DERS was more comprehensive to cover a wider range of emotion regulation strategies including the clarity of emotions, acceptance of emotional experience, impulsion control, goal-directed behavior and flexibility in strategies (Gratz and Roemer, 2004), which were in line with the core of mindfulness concept emphasizing the awareness and acceptance of emotion experience, and the self-control ability. Regarding the emotion regulation process, the ERQ addressed the dynamic process of emotion regulation through measuring two strategies including cognitive reappraisal and expressive suppression (Gross and John, 2003). Other strategies such as the attention deployment proposed in the model are not measured in this scale. In addition, based on the concepts and constructs of DERS and ERQ, emotion regulation in DERS tends to be a capacity of individual’s inherent trait, while emotion regulation in ERP seems to emphasize more on a dynamic process. The present results indicated that one’s inherent capacity of emotion regulation measuring by DERS contributed more to the mechanism underlying mindfulness and psychological distress.

The present findings have important practical implications. Based on the our results, dispositional mindfulness appeared to be a protective factor against adolescents’ psychological distress through emotion regulation. Mindfulness-based interventions have been increasingly recognized for their effectiveness in reducing psychological distress across a range of clinical disorders and non-clinical psychological problems (Kabat-Zinn, 2003). Some studies have demonstrated that mindfulness-based programs are effective in facilitating adolescents’ well-being (e.g., Schonert-Reichl and Lawlor, 2010; Kallapiran et al., 2015). Therefore, effective mindfulness-based prevention programs should be taken into consideration to help adolescents improve their emotion regulation ability and reduce psychological distress.

## Limitations and Future Research Directions

Several limitations should be noted when generalizing the results of the present study. First, although the model of this study was built on existing theory and literature, future studies could incorporate a longitudinal design to confirm the causal relationships between variables. Also, intervention studies can also be considered to test whether mindfulness-based programs for adolescents can effectively reduce adolescents’ emotion regulation difficulties and psychological distress. Second, the measures used in the present study were all generated from adolescents’ self-reports. To improve the validity of the findings, different measuring methods and resources should be used such as reports from parents’ perspectives, behavioral measurements, and so forth. Third, although the MASS used in the present study is the most widely used measurement of dispositional mindfulness, it is a single-factor scale. Future research could adopt a multidimensional scale of mindfulness to explore the multi-faceted mindfulness structure in adolescent sample. Fourth, the sample in the present study was mainly based on convenience and voluntary participation. Findings should be generalized with caution. Future research could use random sampling to confirm the results of this study. Fifth, we mainly explored the mechanism underlying the relationship between mindfulness and adolescents’ psychological distress in perspective of emotion regulation. There are some other mechanisms underlying this relationship, such as self-regulation and autonomy (Parto and Besharat, 2011), reduced rumination (Coffey and Hartman, 2008), and attachment style (McDonald et al., 2016). Therefore, more research is still needed to broaden our understandings on the association between mindfulness and psychological distress on the basis of different theories and mechanisms. Last but not the least, due to the rapid changes in the situation of international policy and economy, although it is not the main focus of the current study, it is reasonable to take the global economic crisis into account as one of the potential predictors of psychological distress. Literature has shown significant influence of economic crisis on mental health (Giorgi et al., 2015; Mucci et al., 2016b), it would be interesting to further explore its impact on psychological distress among adolescents as a transition period in life. Arkes (2007) found that economic downturns are related to adolescent alcohol and drug use. An effective intervention such as mindfulness-based program at early stage might prevent the emergence of adolescents’ psychological distress and problem behaviors.

## Conclusion

Adolescents’ with high levels of dispositional mindfulness may lead to lower level of psychological distress including depression, anxiety, and stress. These associations could be explained by comprehensive abilities of emotion regulation, especially Acceptance and Strategies. Moreover, mindfulness might help reduce adolescents’ psychological distress through reducing expressive suppression of emotion experiences. DERS, which aims at measuring the overall capacity of emotion regulation, appears to contribute more to the mechanism underlying adolescents’ mindfulness and psychological distress than ERQ which focuses on the dynamic process of emotion regulation.

## Ethics Statement

The study was approved by the Ethics Committee of the Chinese University of Hong Kong. Written informed consent was obtained from the parents/legal guardians of all participants.

## Author Contributions

YM mainly contributed to the design of the study and writing of the manuscript. SF critically reviewed and revised the manuscript. Both authors were accountable for the final version of the manuscript.

## Conflict of Interest Statement

The authors declare that the research was conducted in the absence of any commercial or financial relationships that could be construed as a potential conflict of interest.
